# Clinical, cytogenetic and molecular study of a case of ring chromosome 10

**DOI:** 10.1186/s13039-015-0124-9

**Published:** 2015-04-21

**Authors:** Živilė Čiuladaitė, Birutė Burnytė, Danutė Vansevičiūtė, Evelina Dagytė, Vaidutis Kučinskas, Algirdas Utkus

**Affiliations:** Department of Human and Medical Genetics, Faculty of Medicine, Vilnius University, Santariškių st. 2, LT-08661 Vilnius, Lithuania; Centre for Medical Genetics, Vilnius University Hospital Santariki Klinikos, Vilnius, Lithuania

**Keywords:** Ring chromosome 10, Dysmorphic features, Bronchial asthma, Array-CGH

## Abstract

Ring chromosome 10 is a rare cytogenetic finding. Only a few cases with molecular cytogenetic definition have been reported. We report here on a child with a ring chromosome 10, which is associated with prenatal and postnatal growth retardation, microcephaly, dysmorphic features, hypotonia, heart defect, severe *pes equinovarus*, and bronchial asthma. The chromosomal aberration was defined by chromosome microarray analysis, which revealed two deletions at 10pter (3.68 Mb) and 10qter (4.26 Mb). The clinical features are very similar to those reported in other clinical cases with ring chromosome 10, excluding bronchial asthma, which has not been previously reported in individuals with ring chromosome 10.

## Background

Constitutional ring chromosomes have been identified for each of the human chromosomes, and overall frequency is estimated at 1 in 30,000 to 60,000 births [1]. Rings result from rare intrachromosomal fusions, although the mechanisms underlying chromosomal ring formation are not completely understood.

Ring chromosome 10 is a rare cytogenetic finding, currently reported in 17 unrelated patients. Common clinical features in these patients include short stature, intellectual disability, microcephaly, facial dysmorphism, and ophthalmologic and urinary tract abnormalities [2]. Clinical features vary, however, depending on the position of the breakpoints and on the level of mosaicism resulting from the unstable nature of the ring upon cell division [3]. Thus, a comprehensive diagnosis of an individual with a ring chromosome requires both a molecular diagnostic approach such as array-CGH and a cytogenetic approach to determine a specific individual diagnosis. Here we describe the clinical features of the patient with the largest apparently stable ring chromosome 10.

## Case presentation

**Figure 1:**
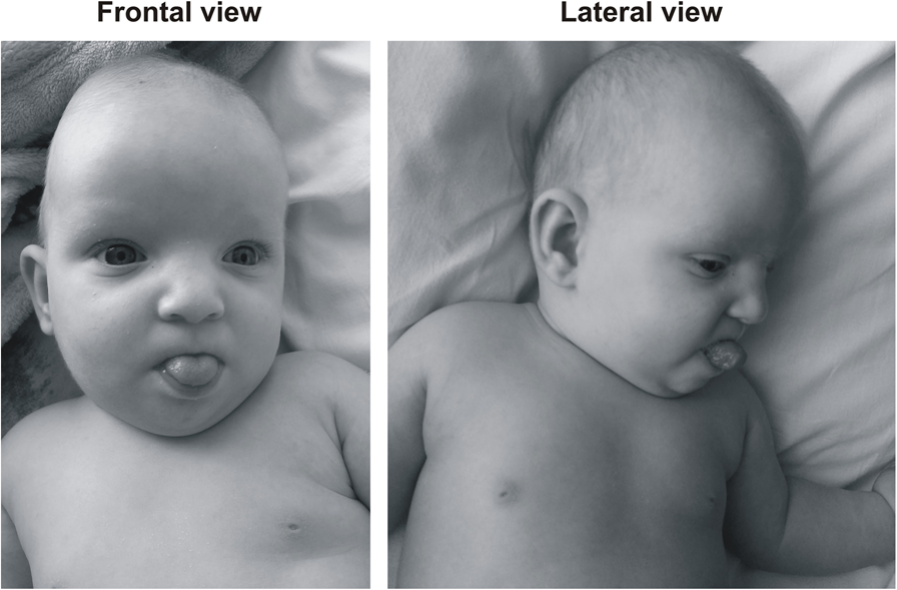
Photographs of the patient at the age of 7 months (frontal and lateral view). Note the downslanting and narrowing of palpebral fissures, broad nasal bridge, stubby nose, smooth philtrum with thin upper lip and everted lower lip, microstomia, low-set ears, and short neck.

The patient is a 13-month-old girl born at 36 weeks of gestation to non-consanguineous and healthy Caucasian parents aged 27 years (mother) and 33 years (father). In utero, intra-uterine growth retardation with meconium staining in the amniotic fluid was observed. She was delivered by elective caesarean section. At birth her weight was 1,600 g (−2.5 SD), her length was 40 cm (-2.5 SD), and the head circumference was 30 cm (−1 SD). Apgar scores were 4–8. Severe congenital *pes equinovarus* was detected from birth. She was also noted to have hypotonia. Shortly after birth, treatment with the Ponseti method was started with surgical correction of the Achilles tendon at 3 months of age. Neurosonoscopy revealed mild widening of the ventricles, but re-evaluation after 1 month was normal. She was found to have normal hearing acuity after birth. A cardiac ultrasound examination showed a large patent *ductus arteriosus*. She had no feeding difficulties. Abdominal organs were without structural abnormalities. At the age of 4 months, bronchial asthma was diagnosed and she has been receiving medical treatment ever since. Examination at the age of 7 months revealed delayed speech and gross motor skills. Her height, weight, and head circumference were significantly less than the 3rd percentile. Furthermore, the patient had dysmorphic features consisting of microcephaly, slight metopic ridge, low-set ears, downslanting and narrowing of palpebral fissures, broad nasal bridge, stubby nose, smooth philtrum with thin upper lip and everted lower lip, microstomia, narrow palate, short neck, inverted and widely-spaced nipples, broad hands, tapering fingers, single palmar crease on the left palm, and broad feet with short toes and small nails (Figure 1). Mild divergent strabismus was documented at that time. Otolaryngological evaluations revealed a deviated septum. Skull roentgenograms revealed no synostosis.

On the last examination at 13 months of age, her development milestones were found to be delayed. She could not sit unsupported and her head control was insufficient. She showed good visual fixation. There was no speech development. Muscle tone was decreased and deep tendon reflexes were normal. She displayed unusual repetitive hand movements, continuously pressing her palms together in the midline and repetitively stroking her thumbs. A brain MRI was declined by her parents. At the age of 7 months, the proband was referred to a clinical geneticist.

### Materials and methods

#### Standard cytogenetics

Cultures of the patient’s peripheral blood were established and harvested according to standard laboratory protocols. Chromosome preparations were treated with trypsin and stained with Giemsa. A total of 30 metaphase cells were analysed at the 550-band resolution level. The karyotypes were described according to the guidelines of the International System for Human Cytogenetic Nomenclature [46,XX,r(10)(p15.1q26.1)]. Additionally, 370 cells were counted to verify ring instability. The parents declined to undergo chromosomal analysis.

#### Molecular cytogenetics

DNA was extracted from the patient’s peripheral white blood cells using the phenol-chloroform extraction method. A subsequent array-comparative genomic hybridisation (array-CGH) test was performed to determine the chromosomal breakpoints of the ring, as well as other possible chromosomal abnormalities that may have been missed by routine G-banded chromosomal analysis. Agilent Human ISCA CGH 180 K microarrays with an average spatial resolution of 25 kb were used in the study (Agilent Technologies, Santa Clara, CA). Genomic DNA from the proband and pooled normal male reference DNA (Agilent Technologies) were digested with Covaris S220 (Life Technologies, Grand Island, NY) and labelled with an Agilent Genomic DNA labelling kit according to the manufacturer’s recommendations. Patient and reference DNA were labelled with Cy5 and Cy3 respectively and were co-hybridised to arrays for 24 h at 67°C in a rotating oven (Agilent technologies) at 20 rpm. The arrays were then washed and scanned with an Agilent Microarray Scanner. Data were extracted using Feature Extraction 10.7.1 software (Agilent Technologies) and analysed using Cytogenomics 2.9.2.4 software (Agilent Technologies). Genomic copy number changes were identified with the assistance of the Aberration Detection Method 2 algorithm with the sensitivity threshold set at 6.0. Copy number changes identified in the samples were evaluated by using the UCSC Genome Browser website (http://genome.ucsc.edu) and the Database of Genomic Variants (http://projects.tcag.ca/variation). The array data was analysed using annotation GRCh37/hg19. The DECIPHER (http://decipher.sanger.ac.uk/) database was used to support genotype-phenotype correlation.

### Results

**Figure 2:**
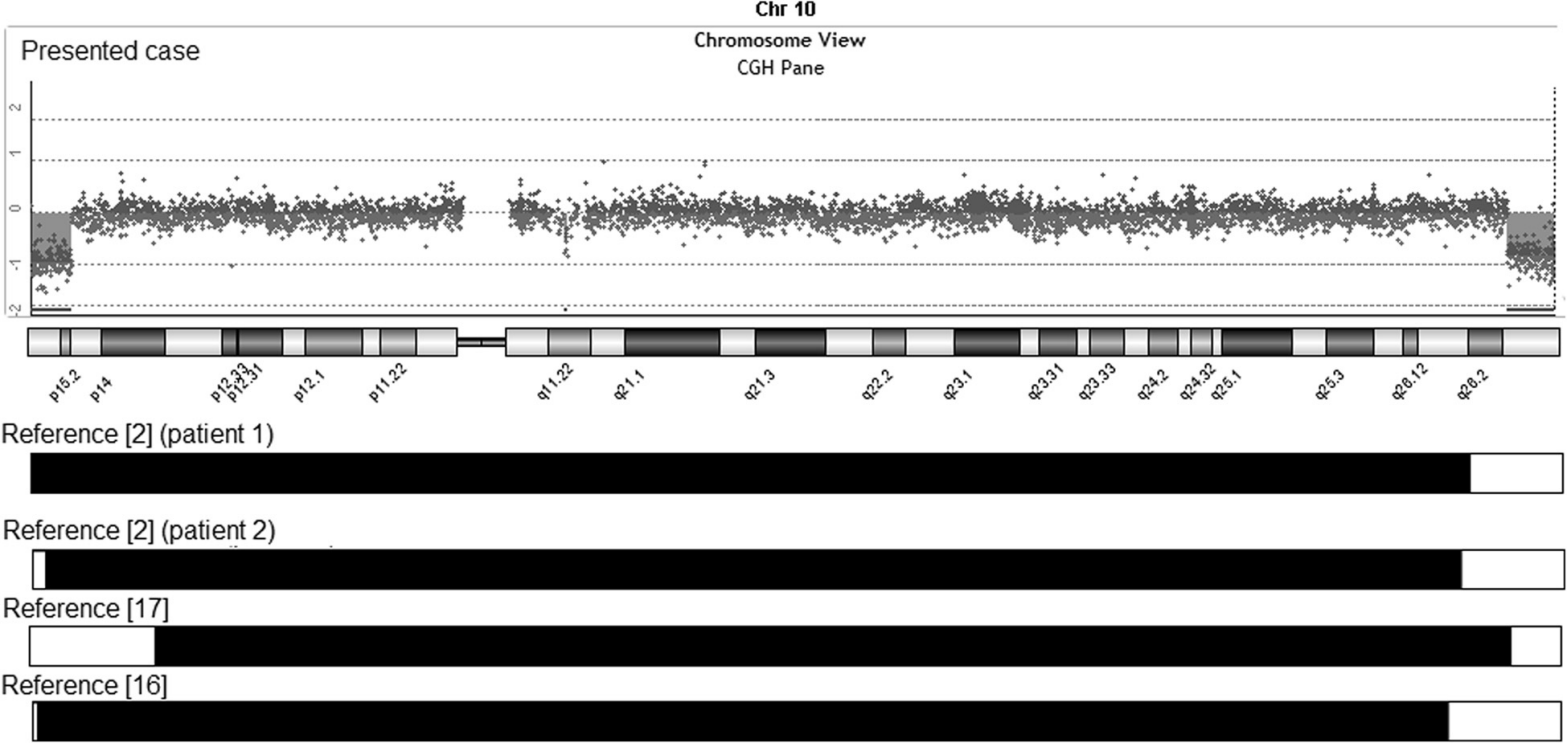
Chromosome 10 array-CGH profile of the patient showing a 3.68 Mb deletion at 10pter and a 4.26 Mb deletion at 10qter. A comparison of the extension of the deletions with previously reported patients with ring chromosome 10 is also shown (white bars).

Cytogenetic analysis revealed an apparently stable non-mosaic ring chromosome 10 [46,XX,r(10) (p15.1q26.1)]. Secondary aberrations (two separate rings and interlocked rings) were found in less than 5% of the mitoses counted, 1.7% and 0.5% respectively. High-resolution breakpoint mapping with a Human ISCA CGH 180 K microarray re-defined the karyotype as 46,XX,r(10)(p15.2q26.3).arr[hg19]10p15.3p15.2(1–3,678,763)×1,10 q26.3(131,276,836–135,534,747)×1, indicating an approximately 3.68 Mb deletion in 10p and a 4.26 Mb deletion in 10q (Figure 2). No other relevant genomic imbalance was found.

### Discussion

Ring chromosome 10 is a rare disorder. Only seventeen cases of ring chromosome 10 have been reported in literature and mostly defined by G banding [3-15] and only four cases with molecular cytogenetic definition [2,16,17]. This is the fifth case with precisely defined r(10) helping to better establish a karyotype-phenotype correlation.

We compared the phenotype and genotype of our patient with previously published patients having precisely defined breakpoints [2,16,17]. The patient we present has the largest ring chromosome 10 reported to date. In all previously reported patients with ring chromosome 10, the breakpoints in 10q are variable but more proximal to the centromere (Figure 2).

Common clinical features in patients with ring chromosome 10 include prenatal and postnatal growth retardation, varying degrees of intellectual disability, microcephaly, and dysmorphic features (broad nasal bridge, strabismus, hypertelorism, low-set malformed ears) [18,19] that are non-specific to distal 10q deletion and are common to many chromosome anomalies. It is unlikely that 3.68 Mb terminal 10p15.2 deletion in the present ring chromosome or smaller deletion in case of ring chromosome reported by Gunnarson *et al.* would make a significant contribution to the phenotype. However the patient with a larger deletion of chromosome 10 short arm has additional clinical features such as *talipes equinovarus*, hepatomegaly, splenomegaly that cause severe phenotype [17].

We also compared the clinical features of our patient with patients from DECIPHER (Table 1). Cases with complex chromosomal rearrangements or with larger deletions than those identified in our patient and cases without a detailed clinical description were excluded from the comparison. Based on comparison of the clinical features of our patient with the clinical features of the patients with ring chromosome and the clinical features of patients with pure terminal deletions of 10p and 10q, the contribution of the terminal deletion 10q to the clinical phenotype of our patient is the most significant.

**Table 1:** **Comparison of clinical features associated with pure 10p deletions and pure 10q deletions published in DECIPHER**

**Deletion interval, hg19**	**Protein coding genes**	**Size (Mb)**	**Phenotypes**						**DECIPHER ID**
			**Developmental delay/ID**	**Facial dysmorphism**	**Hand malformations**	**Cardiac malformations**	**Short stature**	**Epilepsy**	
**10p15**									
10:269607-1380732	*ZMYND11, DIP2C, PRR26, LARP4B, GTPBP4, IDI2, WDR37, ADARB2-AS1*	1.11	+	-	-	-	+	+	1232
10:136361-1758581	*ZMYND11, DIP2C, PRR26, LARP4B, GTPBP4, IDI2, WDR37, ADARB2-AS1*	1.62	+	+	-	-	+	-	2319
10:299304-740247	*ZMYND11, DIP2C, PRR26*	0.44	+	+	+	-	-	-	270190
10:723328-1214416	*DIP2C, PRR26, LARP4B, GTPBP4, IDI2*	0.49	+	-	-	-	-	-	271618
10:148206-2461302	*ZMYND11, DIP2C, PRR26, LARP4B, GTPBP4, IDI2, WDR37, ADARB2-AS1*	2.31	+	-	-	-	-	-	274302
10:158945-313504	*ZMYND11*	0.15	+	+	-	+	+	-	248177
10:148206-1232090	*ZMYND11, DIP2C, PRR26, LARP4B, GTPBP4, IDI2, WDR37, ADARB2-AS1*	1.08	+	-	-	-	-	-	290840
**10q26**									
loss 10:131489998-135390508	*MGMT, EBF3, GLRX3, TCERG1L, PPP2R2D, BNIP3, JAKMIP3, DPYSL4, STK32C, LRRC27, PWWP2B, C10orf91, INPP5A, NKX6-2, C10orf93, GPR123, KNDC1, UTF1, VENTX, ADAM8, TUBGCP2, ZNF511, CALY, PRAP1, C10orf125, ECHS1, PAOX, MTG1, SPRN, CYP2E1, SYCE1, SPRNP1*	3.90	-	+	+	-	-	-	3452
loss 10:135057537-135434113	*ADAM8, TUBGCP2, ZNF511, CALY, PRAP1, C10orf125, ECHS1, PAOX, MTG1, SPRN, CYP2E1, SYCE1, SPRNP1*	0.38	+	-	-	-	-	+	263009
loss 10:135053398-135404523	*VENTX, ADAM8, TUBGCP2, ZNF511, CALY, PRAP1, C10orf125, ECHS1, PAOX, MTG1, SPRN, CYP2E1, SYCE1*	0.35	-	-	-	-	-	-	286726

Cases with larger deletions than those identified in our patient and cases without a detailed clinical description were excluded from the comparison.

ID, intellectual disability.

The study of patients with a distal pure 10q deletion has revealed the existence of a minimal critical region (MCR), which was recently assigned by Yatsenko et al. [20] to an approximately 600 kb segment in the distal part of chromosome 10, which encompasses two annotated genes, *C10ORF90* (chromosome 10 open reading frame 90) and *DOCK1* (dedicator of cytokinesis 1). We predict that the overlapping phenotype of pure 10q deletions at 10q26.2 region could be caused by haploinsufficiency of one or more genes or position effect, since 10q26.3 deletion detected in our patient does not involve these genes.

The deleted region 10q26.3, 4.26 Mb in size, contains 31 protein coding genes of which *PPP2R2D* (protein phosphatase 2, regulatory subunit B, delta), *JAKMIP3*(Janus kinase and microtubule interacting protein 3), *DPYSL4* (dihydropyrimidinase-like 4), *INPP5A* (inositol polyphosphate-5-phosphatase), *GPR123* (G protein-coupled receptor 123), *GLRX3* (glutaredoxin 3) and *ADAM8* (ADAM metallopeptidase domain 8) could be considered important contributors to the clinical phenotype. Based upon function and high expression in the brain [http://www.proteinatlas.org/], we suggest that the haploinsuffiency of *PPP2R2D*, *JAKMIP3*, *DPYSL4*, and *GPR123* could play significant roles in neurodevelopmental delay. PPP2R2D is essential for many signal transduction pathways [21]. JAKMIP3 is associated with caveolin-1, which recruits synaptic components and regulates the signal transduction of a variety of neurotransmitter and neurotrophic receptors in the central nervous system (CNS) [22]. The collapsin response mediator protein encoded by *DPYSL4* is thought to be involved in semathorin-induced growth cone collapse during neural development. Down-regulation of *DPYSL4* expression using siRNA shows an early increase in neurite outgrowth, further supporting the idea that DPYSL4 inhibits microtubule polymerisation and neurite outgrowth [23]. The CNS-specific expression of *GPR123*, together with the high sequence conservation between the vertebrate sequences investigated, indicate that GPR123 may have an important role in the regulation of neuronal signal transduction [24].

Craniofacial dysmorphisms, foot abnormalities, and short stature could be attributed to the loss of the *GLRX3* gene. Although growth delay is usually associated with the ring chromosome of any autosome, possibly due to ring instability [1], stature might also correlate with the haploinsuffiency of genes that encode protein and play a role in cell growth. The ubiquitous expression of *Glrx3* in mouse embryos and tissues indicates that *Glrx3* is required for cell growth, organ development, and normal metabolism during growth and development [25]. Thus, deletion of *GLRX3* might influence the severity of growth delay. The patent *ductus arteriosus* could be associated with the haploinsuffiency of *DPYSL4*, *PPP2R2D*, and *INPP5A*, the expression of which is predominant in the heart [http://www.proteinatlas.org/].

The second deleted region, 10p15.2-pter, could also contribute to the observed phenotype. The 10p15.3p15.2 deleted region contains 11 protein-coding genes (*TUBB8* (tubulin, beta 8 class VIII), *ZMYND11* (zinc finger, MYND-type containing 11), *DIP2C* (DIP2 disco-interacting protein 2 homolog C), *PRR26* (proline rich 26), *LARP4B* (La ribonucleoprotein domain family, member 4B), *GTPBP4* (GTP binding protein 4), *IDI2* (isopentenyl-diphosphate delta isomerase 2), *WDR37* (WD repeat domain 37), *ADARB2-AS1* (ADARB2 antisense RNA 1), *PFKP* (phosphofructokinase, platelet), *PITRM1* (pitrilysin metallopeptidase 1), from which *DIP2C* and *ZMYND11* could be considered important contributors to growth delay, since they were most commonly deleted in DECIPHER patients with common clinical features, short stature and microcephaly (Table 1). *ZMYND11* [26] and *DIP2C* [27] are expressed in various tissues, including the brain, but little is known about their function. Gunnarson *et al.* stated that loss of 10p15.3 region including *ZMYND11* would contribute little to the clinical phenotype because of significant larger terminal deletion at 10q [16]. However *ZMYND11* was suggested by DeScipio *et al.* as a main contributor to the clinical features associated with 10p15 deletions based on genotype-phenotype of the cases with isolated 10p deletions [28].

In addition to the clinical features commonly found in patients with ring chromosome, bronchial asthma was present in our patient. This clinical feature had not previously been reported in patients with ring chromosome 10. The *ADAM8* mapped at 10q26 could be involved in asthma pathogenesis. In humans, *ADAM8* is expressed by most leukocytes [29,30], lung epithelial cells [31], and osteoclasts [32]. More recently, ADAM8 has been strongly associated with allergic airway inflammation (AAI) in humans and mice, and additional studies of ADAM8 are beginning to shed light on its roles in asthma pathogenesis [33].

## Conclusions

The case reported here together with clinical and molecular findings, compared to previously published cases, highlights the importance of microarray analysis for patients with ring chromosomes, since it helps to delineate specific phenotypes. We were able to determine the gene content of the regions and make karyotype-phenotype correlations after having refined the exact breakpoints of the deletions. Further functional studies of candidate genes are needed to prove biological significance in growth and development.

## Consent

This case report is presented with the informed consent of the patient’s parents. A copy of the written consent is available for review by the editor-in-chief of this journal.

## Abbreviations

Array-CGH: Array based comparative genomic hybridisation
CNS: Central nervous system

## References

[1] Kosztolányi G (2009). The genetics and clinical characteristics of constitutional ring chromosomes. J Assoc Genet Technol.

[2] Guilherme RS, Kim CA, Alonso LG, Honjo RS, Meloni VA, Christofolini DM (2013). Ring chromosome 10: report on two patients and review of the literature. J Appl Genet.

[3] Nakai H, Adachi M, Katsushima N, Yamazaki N, Sakamoto M, Tada K (1983). Ring chromosome 10 and its clinical features. J Med Genet.

[4] Lansky S, Daniel W, Fleizar K (1977). Physical retardation associated with ring chromosome mosaicism: 46, XX, r(10)/45, XX,-10. J Med Genet.

[5] Fryns P, De Boeck K, Jaken J, van den Berg H (1978). Malformative syndrome associated with a ring 10 chromosome and translocated 10q/19 chromosome. Hum Genet.

[6] Sparkes RS, Ling SM, Muller H (1978). Ring 10 chromosome: 46, XX, r(10)(p15q26). Hum Genet.

[7] Simoni G, Rossella F, Dalpra L, Visconi G, Piria-Schwaz C (1979). Ring chromosome 10 associated with multiple congenital malformations. Hum Genet.

[8] Tsukino R, Tsuda N, Dezawa T, Ishii T, Koike M (1980). Ring chromosome 10: 46, XX, r(10)(p15q26). J Med Genet.

[9] Michels VV, Driscoll DJ, Ledbetter DH, Riccardi VM (1981). Phenotype associated with ring 10 chromosome: report of patient and review of literature. Am J Med Genet.

[10] Serville F, Briault R, Taillemite JL, Despoisse S, Cotoni P, Broustet A (1982). Ring chromosome 10: 46, XX, r(10)(p15q26). Ann Genet.

[11] Kondo I, Shimakura Y, Hirano T, Kaneko M, Yabuta K (1984). Ring chromosome 10 syndrome: case report and the possibility of clinical diagnosis. Clin Genet.

[12] Kishi K, Ikeuchi T, Yamamoto K, Tonomura A, Sakurada N, Satoh Y (1985). Report of a patient with a ring chromosome 10: mos45, XY,-10/46, XY/46, XY, r(10)(p15.3q26.3). Jinrui Idengaku Zasshi.

[13] Higashi K, Sarashina N, Okamoto T, Matsuki C, Heim S (1992). Supernumerary ring marker chromosome as a secondary rearrangement in a parapharyngeal lipoma with t(10;12)(q25;q15) as the primary karyotypic abnormality. Cancer Genet Cytogenet.

[14] Calabrese G, Franchi PG, Stuppia L, Mingarelli R, Rossi C, Ramenghi L (1994). A newborn with ring chromosome 10, aganglionic megacolon, and renal hypoplasia. J Med Genet.

[15] Concolino D, Iembo MA, Moricca MT, Strisciuglio P, Marotta R, Rossi E (2003). Ring chromosome 10 (p15q26) in a patient with unipolar affective disorder, multiple minor anomalies, and mental retardation. Am J Med Genet.

[16] Gunnarsson C, Graffmann B, Jonasson J (2009). Chromosome r(10)(p15.3q26.12) in a newborn child: case report. Mol Cytogenet.

[17] Christopoulou G, Tzetis M, Konstantinidou AE, Tsezou A, Kanavakis E, Kitsiou-Tzeli S (2012). Clinical and molecular description of a fetus in prenatal diagnosis with a rare de novo ring 10 and deletions of 12.59 Mb in 10p15.3–p14 and 4.22 Mb in 10q26.3. Eur J Med Genet.

[18] Wulfsberg EA, Weaver RP, Cunniff CM, Jones MC, Jones KL (1989). Chromosome 10qter deletion syndrome: A review and report of three new cases. Am J Med Genet.

[19] Plaisancie J, Bouneau L, Cances C, Garnier C, Benesteau J, Leonard S (2014). Distal 10q monosomy: New evidence for a neurobehavioral condition?. Eur J Med Genet.

[20] Yatsenko SA, Kruer MC, Bader PI, Corzo D, Schuette J, Keegan CE (2009). Identification of critical regions for clinical features of distal 10q deletion sindrome. Clin Genet.

[21] Batut J, Schmierer B, Cao J, Raftery LA, Hill CS, Howell M (2008). Two highly related regulatory subunits of PP2A exert opposite effects on TGF-beta/Activin/Nodal signalling. Development.

[22] Stern CM, Mermelstein PG (2010). Caveolin regulation of neuronal intracellular signaling. Cell Mol Life Sci.

[23] Aylsworth A, Jiang SX, Desbois A, Hou ST (2009). Characterization of the role of full-length CRMP3 and its calpain-cleaved product in inhibiting microtubule polymerization and neurite outgrowth. Exp Cell Res.

[24] Lagerström MC, Rabe N, Haitina T, Kalnina I, Hellström AR, Klovins J (2007). The evolutionary history and tissue mapping of GPR123: specific CNS expression pattern predominantly in thalamic nuclei and regions containing large pyramidal cells. J Neurochem.

[25] Cheng NH, Zhang W, Chen WQ, Jin J, Cui X, Butte NF (2011). A mammalian monothiol glutaredoxin, Grx3, is critical for cell cycle progression during embryogenesis. FEBS J.

[26] Kurozumi K, Nishita M, Yamaguchi K, Fujita T, Ueno N, Shibuya H (1998). BRAM1, a BMP receptorassociated molecule involved in BMP signaling. Genes Cells.

[27] Nagase T, Ishikawa K, Suyama M, Kikuno R, Hirosawa M, Miyajima N (1999). The complete sequences of 100 new cDNA clones from brain which code for large proteins in vitro. DNA Res.

[28] DeScipio C, Conlin L, Rosenfeld J, Tepperberg J, Pasion R, Patel A (2012). Subtelomeric deletion of chromosome 10p15.3: Clinical findings and molecular cytogenetic characterization. Am J Med Genet A.

[29] Richens J, Fairclough L, Ghaemmaghami AM, Mahdavi J, Shakib F, Sewell HF (2007). The detection of ADAM8 protein on cells of the human immune system and the demonstration of its expression on peripheral blood B cells, dendritic cells and monocyte subsets. Immunobiology.

[30] Gomez-Gaviro M, Dominguez-Luis M, Canchado J, Calafat J, Janssen H, Lara-Pezzi E (2007). Expression and regulation of the metalloproteinase ADAM-8 during human neutrophil pathophysiological activation and its catalytic activity on L-selectin shedding. J Immunol.

[31] Foley SC, Mogas AK, Olivenstein R, Fiset PO, Chakir J, Bourbeau J (2007). Increased expression of *ADAM33* and *ADAM8* with disease progression in asthma. J Allergy Clin Immunol.

[32] Ainola M, Li TF, Mandelin J, Hukkanen M, Choi SJ, Salo J (2008). Involvement of ADAM8 in osteoclastogenesis and pathological bone destruction. Ann Rheum Dis.

[33] Knolle MD, Owen CA (2009). ADAM8: A new therapeutic target for asthma. Expert Opin Ther Targets.

